# Supplementary material for: Self-Reporting Theranostic: Nano Tool for Arterial Thrombosis
Source: Bioengineering (Basel). 2023 Aug 29;10(9):1020. doi: 10.3390/bioengineering10091020 (PMC10525380; doi:10.3390/bioengineering10091020)
Supplement: Supplementary file 1 [file bioengineering-10-01020-s001.zip › bioengineering-2268127-supplementary.pdf]

bioengineering

Supplementary Materials

Self-Reporting Theranostic: Nano Tool for Arterial Thrombosis

Suryyani Deb [1,*], Mohammad Azharuddin [2], Sofia Ramström [3], Kanjaksha Ghosh [4], Santiswarup Singha [5], Thobias Romu [6] and Hirak Kumar Patra [7,*]

1 Department of Biotechnology, Maulana Abul Kalam Azad University of Technology, Haringhata, West Bengal 741249, India
2 Cambridge Display Technology Ltd., Huntingdon, Cambridgeshire PE29 2XG, UK
3 School of Medical Sciences, Orebro University, Örebro SE-701 82, Sweden; sofia.ramstrom@oru.se
4 National Institute of Immunohaematology, KEM Hospital Campus, Mumbai, Maharashtra 400012, India
5 National Institute of Immunology, Aruna Asaf Ali Marg, New Delhi 110067, India; santiswarup@nii.ac.in
6 Centre for Medical Image Science and Visualization (CMIV), Department of Biomedical Engineering (IMT), Linköping University, 581 85 Linköping, Sweden
7 Department of Surgical Biotechnology, UCL Division of Surgery and interventional Sciences, University College London, London NW3 2PF, UK
* Correspondence: suryyani.deb@makautwb.ac.in (S.D.); hirak.patra@ucl.ac.uk (H.K.P.); Tel.: +91-9874334818 (S.D.); +44-2031085658 (H.K.P.)

FTIR:

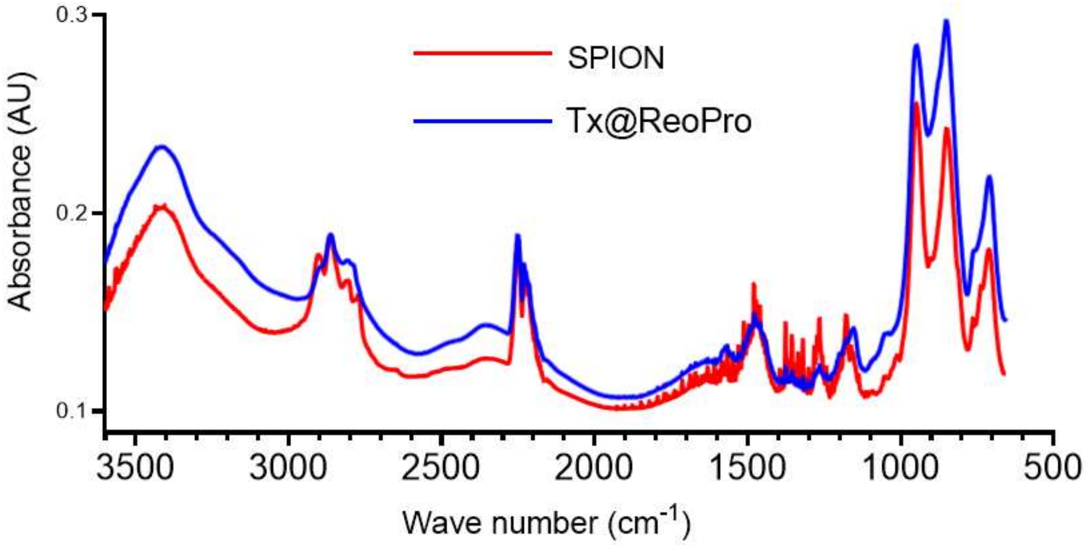

Figure S1. Fourier transform infrared (FTIR) spectroscopy shows change in spectral pattern of SPION after conjugation with ReoPro. The blue line represents SPION, and red line represents ReoPro conjugated SPION (TX@ReoPro).

Light Transmission Aggregometry:

Optical or light transmission Born platelet aggregometry is considered to be the gold standard method for measuring platelet aggregation with platelet-rich plasma (PRP). The percentage (%) of light transmission is evaluated as platelet aggregation when agonists are added to the PRP. Limited light can pass through the colloidal PRP when platelets are in their resting phase, and this is set as baseline or 0% aggregation. After the addition of the agonist, platelets get aggregated with time and settle down due to gravitational force. This leads to an increase in light transmitted through the plasma, which indicates the degree of platelet aggregation.

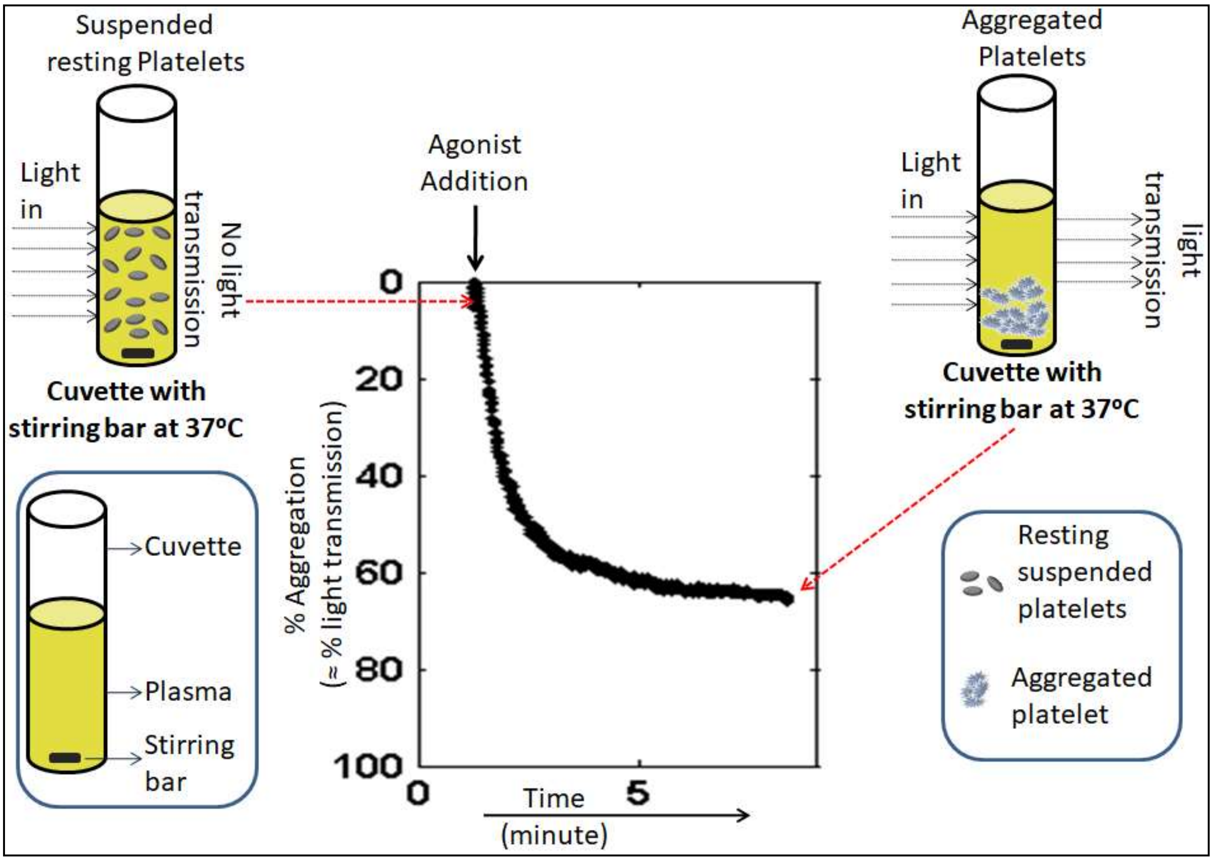

Figure S2A: Schematic diagram of light transmission platelet aggregometry.

Light Transmission Aggregometry: Dose Response:

The concentration-dependent inhibitory effect of Tx@ReoPro on platelet light transmission aggregation is shown below. The concentration of the drug (ReoPro) in Tx@ReoPro varied from 20 µg/ml to 80µg/ml, while the agonist concentration of ADP used to activate platelets remained at 6 µM in all experiments.

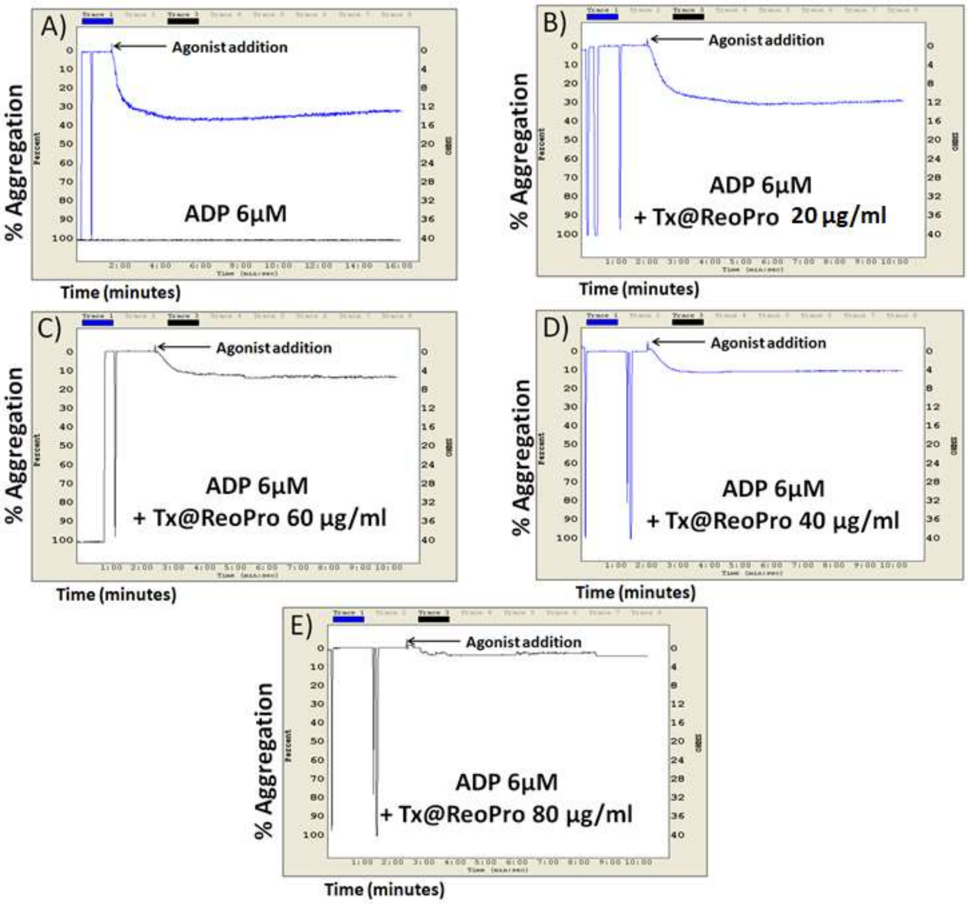

Figure S2B: Effect of SPION and Tx@ReoPro on light transmission platelet aggregation when activated with ADP (6 µM) as agonist. The aggregation was monitored for 10 minutes. Platelet aggregation gradually decreased with the increase in concentration of the Tx@ReoPro.

Light Transmission Aggregometry: Control response:

In this study, the platelet light transmission aggregation was studied with SPION and observed to be almost similar to when activated with ADP 6 µM alone. Therefore, it can be said that SPION did not show any inhibitory effect on platelet aggregation. In the presence of Tx@ReoPro, the aggregation was much lower and comparable with platelet aggregation in the presence of pure ReoPro in the same concentration (80µg/ml). This confirms that Tx@ReoPro-induced inhibition of aggregation is due to the functionalized drug and that ReoPro retains its full activity after the conjugation.

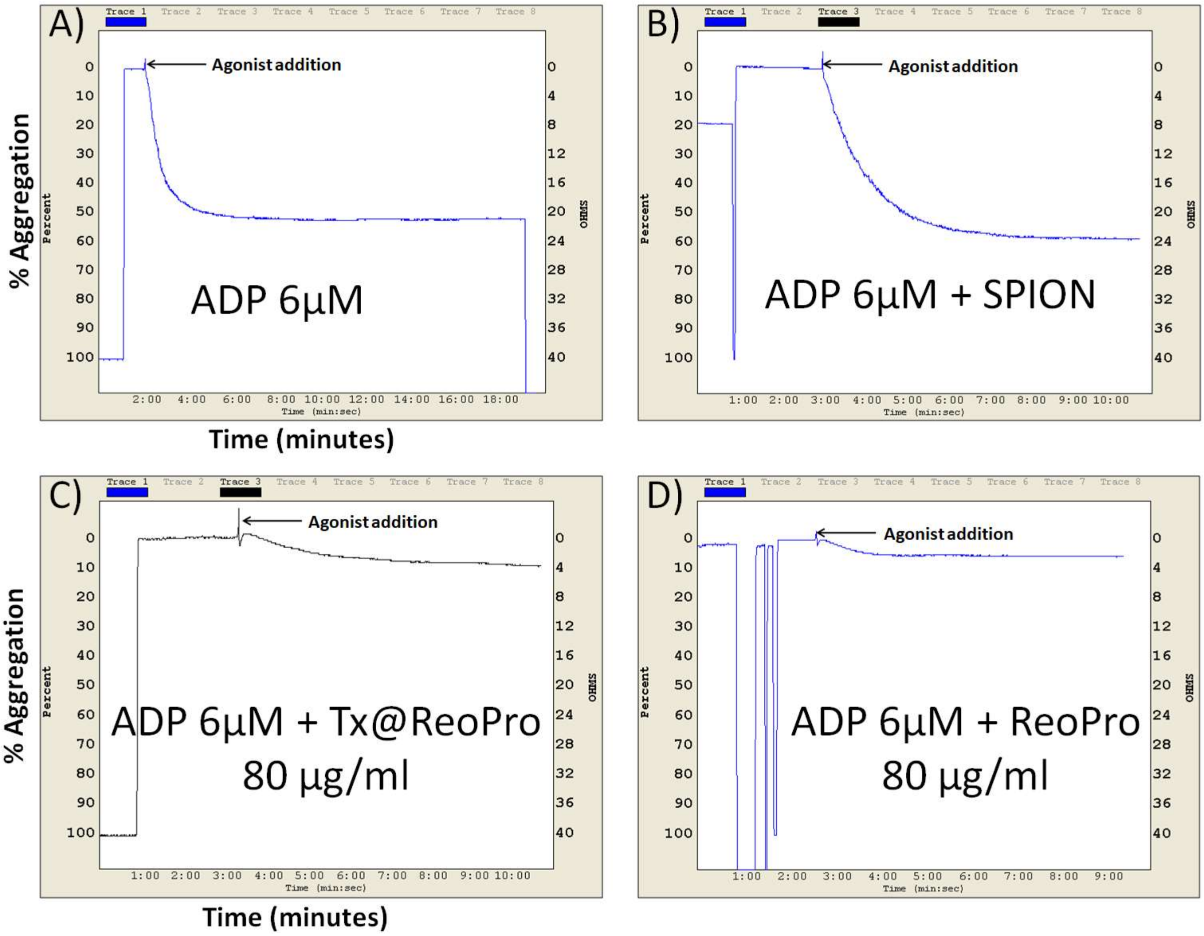

Figure S3. Effect of SPION, Tx@ReoPro, and ReoPro alone on platelets, was studied through light transmission aggregometry, in presence of ADP (6 µM) as agonist.

Magnetic Force Microscope Imaging:

In the standard tapping mode operation, the probe cantilever is set to oscillate at its resonance frequency, f0. As the probe sweeps across the surface, it "feels" the surface by tapping the surface to produce an AFM image of the physical surface. On the return trip across the surface on the same track as before, it scans the same surface, hence giving the image. This is called "trace and retrace". In the case of MFM, which is an advanced mode of tapping mode, the tool is set to its "Lift Mode" operation, so that the probe tip travels above the surface by a set distance, following the height topography from the memory of the just measured physical profile. When the magnetized probe tip "feels" a change in the magnetic field strength rising from the surface, the cantilever oscillation frequency shifts a small amount, perhaps 1 – 50 Hz in magnitude, giving an MFM image.

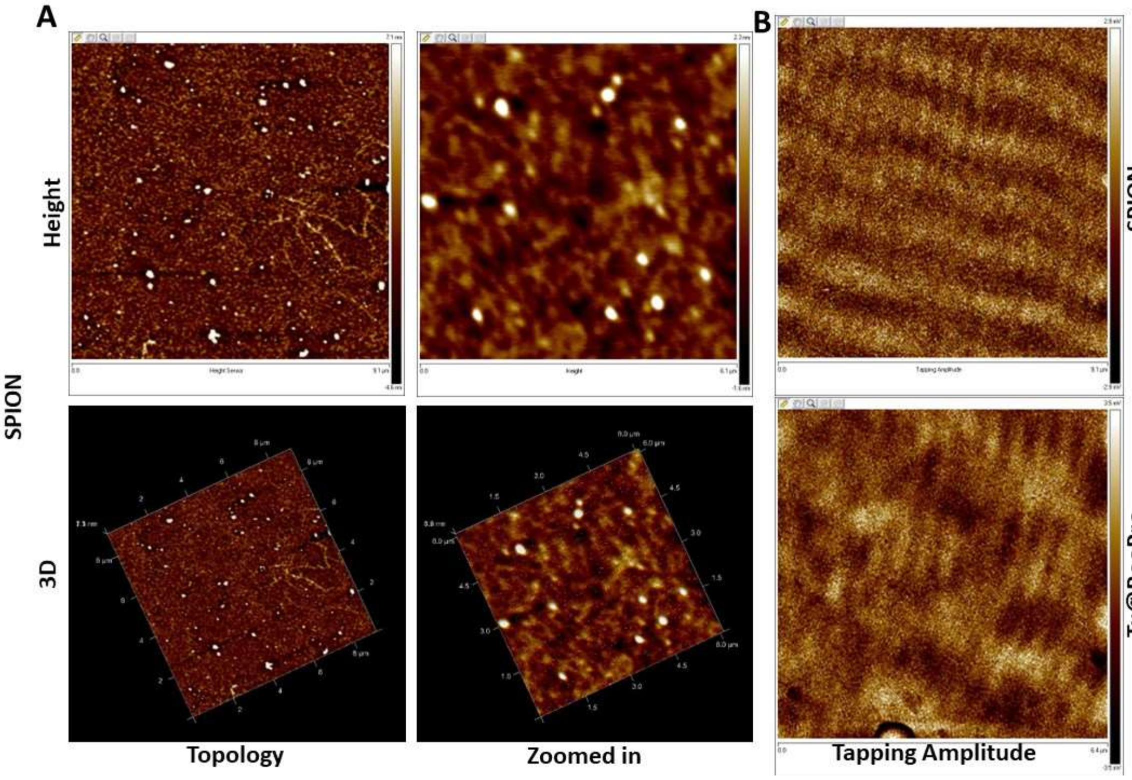

Figure S4. Shows magnetic force microscopy (MFM) image of SPION and Tx@ReoPro. (**A**) Represents the topology of SPION in normal, three-dimensional, and zoomed-in conditions. (**B**) Represents the tapping amplitude of both SPION and Tx@ReoPro.

Flow cytometry: functional validation of individual components:

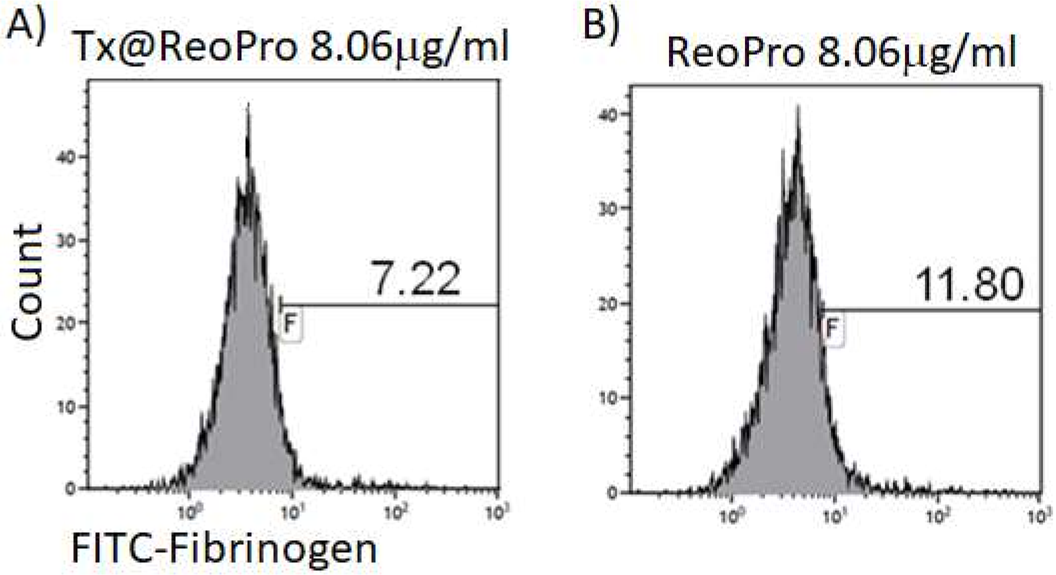

Figure S5. Flow cytometric data showing that inhibition of fibrinogen binding to GPIIbIIIa is more pronounced when ReoPro is conjugated with SPION (Tx@ReoPro) (**A**) than ReoPro (**B**) alone in the same concentration (8.06μg/ml). F is representing percentage of population.

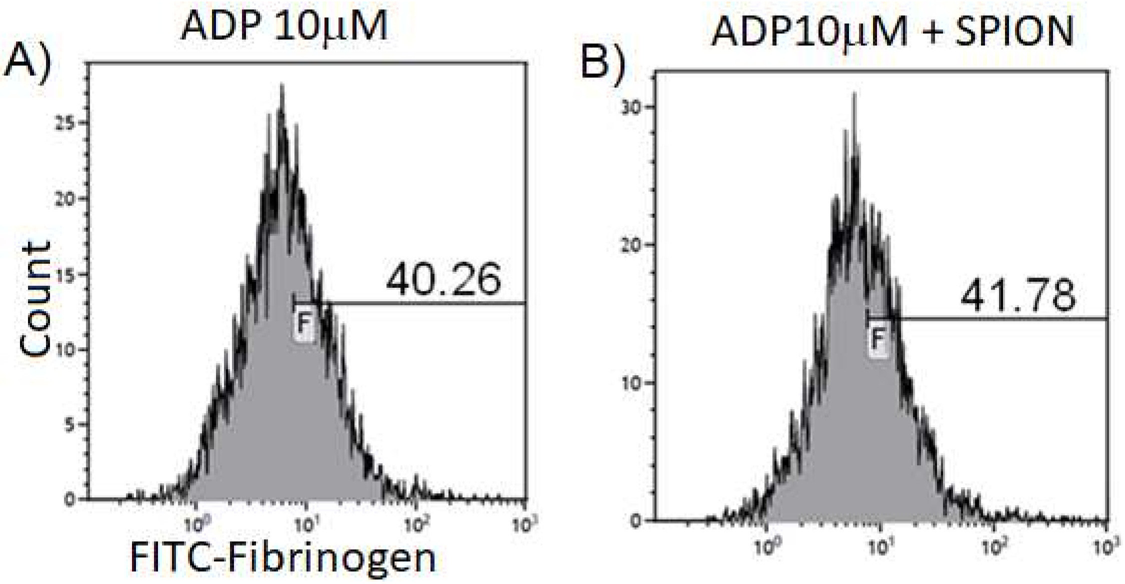

Figure S6. Flow cytometric data showing that fibrinogen binding to GPIIbIIIa is similar with and without SPION. Platelets are activated with ADP 10μM in absence (**A**) and presence of SPION (**B**). Here F is representing percentage of population.
